# Benefits of Quercetin on Glycated Hemoglobin, Blood Pressure, PiKo-6 Readings, Night-Time Sleep, Anxiety, and Quality of Life in Patients with Type 2 Diabetes Mellitus: A Randomized Controlled Trial

**DOI:** 10.3390/jcm13123504

**Published:** 2024-06-15

**Authors:** Aikaterini E. Mantadaki, Manolis Linardakis, Maria Tsakiri, Stella Baliou, Persefoni Fragkiadaki, Elena Vakonaki, Manolis N. Tzatzarakis, Aristidis Tsatsakis, Emmanouil K. Symvoulakis

**Affiliations:** 1 Clinic of Social and Family Medicine, Department of Social Medicine, School of Medicine, University of Crete, 70013 Heraklion, Greece; linman@med.uoc.gr (M.L.); esymvoulakis@uoc.gr (E.K.S.); 2 Iatrica, Local Unit of Lab Analysis and Diagnostics Network, 71303 Heraklion, Greece; tsakirhmaria@gmail.com; 3 Laboratory of Toxicology, Medical School, University of Crete, 71003 Heraklion, Greece or stellabaliou@gmail.com (S.B.); persefoni.f@gmail.com (P.F.); evakonaki@gmail.com (E.V.); tzatzarakis@med.uoc.gr (M.N.T.); tsatsaka@uoc.gr (A.T.)

**Keywords:** type 2 diabetes mellitus, type 2 diabetes, nutraceutical, quercetin, randomized trial, nutritherapy, hypoglycemic effect, phytotherapy, diabetes supportive care, integrated care

## Abstract

**Background:** Diabetes is a rapidly growing global morbidity issue with high prevalence, and the associated dysglycemia leads to complications. Patients with type 2 diabetes mellitus (T2DM) often experience elevated anxiety levels, affecting their quality of life and diabetes management. This study investigated quercetin, a nutraceutical and potential senolytic with antioxidant activity, to detect its possible positive effect on the bio-clinical measurements and routine health of patients with T2DM. **Methods:** This prospective randomized controlled trial (RCT) investigated the clinical usefulness of quercetin in patients with T2DM receiving non-insulin medications. One hundred participants were stratified by age and sex (1:1) and randomized to control (n = 50) or intervention (n = 50) groups. The control received standard care only, while the intervention received 500 mg quercetin daily for 12 weeks, followed by an 8-week washout and a final consecutive 12-week supplementation period (total: 32 weeks), as adjunct to their usual care. Comprehensive health assessments, including blood analyses, were conducted at baseline and study termination. Quality of life and anxiety were assessed using the 36-item Short Form Health Survey (SF-36) and Short Anxiety Screening Test (SAST-10). **Results:** Eighty-eight patients with T2DM concluded the trial. Compared with the control, glycated hemoglobin (HbA1c) levels showed a significant decrease (Δ%-change: −4.0% vs. 0.1%, *p* = 0.011). Quercetin also significantly improved PiKo-6 readings (FEV_1_: 5.6% vs. −1.5%, *p* = 0.002), systolic blood pressure (−5.0% vs. −0.2%, *p* = 0.029), night-time sleep (11.6% vs. −7.3%, *p* < 0.001), anxiety levels (SAST-10) (−26.2% vs. 3.3%, *p* < 0.001), and quality of life (SF-36) (both physical and mental components, *p* < 0.001). **Conclusions:** Based on the current open-label study, quercetin appears to be a promising supplement for T2DM, providing lifestyle and care support. Further research is warranted to shift this potential from clinical usefulness and feasibility to multidisciplinary evidence.

## 1. Introduction

Diabetes constitutes a significant and progressively escalating global health challenge, affecting hundreds of millions of individuals and exerting substantial strain on healthcare systems worldwide [1]. Projections indicate a significant rise in its prevalence within the coming decades [2]. Global population aging significantly contributes to the surging incidence of diabetes worldwide [3]. This initially silent, chronic condition is characterized by dysglycemia, a state of impaired blood sugar regulation, which leads to imbalances between free radical and antioxidant mechanisms [4,5,6]. This imbalance, often secondary to hyperglycemia, contributes to the development of a diverse range of diabetic complications [6]. Type 2 diabetes mellitus (T2DM) accounts for roughly 90–95% of all diabetes cases [7].

Importantly, cellular senescence is a hallmark of T2DM, driven primarily by hyperglycemia and contributing to both compromised cellular responsiveness to insulin signaling and inadequate insulin production by pancreatic β-cells [8,9]. Therefore, aging underlies T2DM in multiple ways, including a reduction in functional β-cell mass and buildup of senescent cells that release inflammatory factors [9]. This chronic inflammation negatively impacts insulin-sensitive tissues and further impairs insulin production, exacerbating diabetic conditions. Building upon this concept, it is crucial to highlight the potential of senolysis, the targeted ablation of senescent cells, as a novel therapeutic strategy for age-related pathologies such as diabetes, potentially targeting disease progression [9]. In addition, maintaining glycemic control serves as the cornerstone of T2DM management, significantly impacting the progression of complications [10]. Researchers and healthcare professionals, in view of the exceedance of former prevalence estimates, are actively engaged in the implementation of effective strategies to optimize diabetes management. These focus on glycemic control and on the optimization of cardiovascular parameters, targeting the prevention of the substantial complications associated with this widespread disease [11].

The rigorous management of key physiological parameters (glycemic, lipid, and blood pressure control) remains crucial for mitigating and decelerating diabetes-related complications [11]. Patients with T2DM are strongly encouraged to follow lifestyle modifications, including dietary adjustments and regular physical exercise, to improve metabolic health [11]. Indeed, this complex metabolic disorder, accompanied by extensive management weaknesses and rising to pandemic levels, necessitates continuous medication and lifestyle adjustments for patients [12]. Furthermore, healthcare providers managing patients with type 2 diabetes should also be aware of potential cardiovascular (CV) complications associated with the accelerated progression of atherosclerosis in case of non-optimal control [10,13].

In general, there is a paucity of trials proving the benefits of nutraceuticals with well-established safety and efficacy profiles. Intriguingly, a widespread use of nutritional supplements in patient groups is prevalent [14] and is often motivated by the misconception that these therapies are inherently safe due to originating from ‘natural’ sources [15]. Moreover, treatment-resistant T2DM, despite combination pharmacotherapy, poses a significant clinical challenge [16], and there is a risk of harmful self-care decisions or non-evidence-based self-choices.

Research supports the pivotal role of oxidative stress in the pathogenesis and progression of diabetes and its sequelae [6,17], triggered by an increased production of reactive oxygen species (ROS), chronic hyperglycemia, and impaired mitochondrial function [6]. This imbalance between ROS and antioxidant dynamics culminates in cellular damage, dysregulated glucose homeostasis, and extensive detriment of organs and tissues [6], prompting explorations of the therapeutic potential of antioxidants [18]. Thus, several medicinal herbal remedies, including berberine [19], silymarin [20] and curcumin [12], among others, have been investigated for their potential in managing diabetic outcomes, although evidence on their safety and efficacy remains scarce [11]. Studies have demonstrated that medicinal plants show some activity of lowering blood glucose levels through mechanisms such as stimulating insulin production [21], improving insulin sensitivity [22], and inhibiting the intestinal enzyme amylase [23].

Quercetin, a naturally occurring flavonoid, features two aromatic rings linked by a bridging heterocyclic unit, along with multiple hydroxyl substituents [24]. This nutraceutical has attracted significant interest due to its presumed pleiotropic therapeutic properties. Emerging research also suggests this phytotherapeutic substance may possess senolytic properties, potentially impacting age-associated diseases like diabetes [25,26]. While it can be found in various plant-based foods [27,28,29], it is typically present in low concentrations as a secondary plant metabolite, rendering its natural concentration in foods limited compared to the commercially available dietary supplements [30]. 

This bioactive botanical displays potent antioxidant and anti-inflammatory activities proven by in vivo laboratory models [13]. Beyond the in vivo-demonstrated vasodilatory [31], antiplatelet [32], and potentially cancer-preventive effects [33], quercetin could also interact with critical cellular signaling pathways [34]. However, translating these findings to human and animal subjects has yielded inconsistent results [13]. Intriguingly, Liu et al. (2006) reported improved cognitive function in metabolically aged mice after quercetin supplementation [35], and a recent double-blind randomized controlled trial supported a similar effect and working memory improvement following supplementation with a quercetin-fortified functional food, hinting at a promising anti-aging effect [36]. 

Quercetin is Generally Recognized as Safe (GRAS) [37]. A randomized controlled trial (RCT) substantiated this favorable safety profile, with limited adverse effects reported at doses of up to 1000 mg daily for 12 weeks [38]. Furthermore, in a phase 1 dose-escalation study involving patients with hepatitis C, quercetin was satisfactorily tolerated in doses up to 5000 mg/day for 28 days, suggesting the maximum tolerated dose (MTD) exceeds this level [39]. Notably, quercetin supplementation does not appear to interfere with the bioavailability of anti-diabetic medications like pioglitazone [40] or rosiglitazone [41]. While published human trials indicate that adverse effects associated with quercetin are rare, mild, and often linked to higher doses, dedicated safety studies in fragile T2DM population groups are lacking. On this premise, and to address this gap, we conducted an interim investigation in a randomized subsample and observed no clinical issues [42], leading to the safe conclusion of the entire study period, the results of which will be presented shortly. 

Additionally, in a recent T2DM model, mice fed quercetin-supplemented diets exhibited dose-dependent reductions in plasma glucose [43]. Besides the scientific interest regarding the potential glucose-lowering effects of antioxidants and the concurrent investigation of hypoglycemic actions of different phytochemical compounds, to the best of our knowledge, only two human studies have probed into the anti-diabetic effect of quercetin on glycated hemoglobin (HbA1c), the most reliable biomarker reflecting long-term average blood glucose levels over the previous trimester [44,45]. 

Not to mention, the potential effects of quercetin on lung function were only recently assessed in humans in a single two-center pilot study involving 14 patients with Idiopathic Pulmonary Fibrosis (IPF) [46]. Findings from the mentioned study, highlighting some premises on physical function, anti-inflammatory and senolytic benefits [46], enhanced our research interest in designing a trial among patients with T2DM. 

Patients with diabetes frequently experience high anxiety levels, which adversely interfere with their quality of life (QoL). This psychological burden further compounds the complexities of diabetes management and potentially contributes to the development of complications [47,48]. While medications targeting anxiety may be prescribed for patients with T2DM [49], thoughts about side effects or long-term intake can compromise treatment adherence and ultimately hinder effective diabetes control [50]. Compared to conventional anti-anxiety drugs, the widely accessible and presumptively minimally addictive nutraceuticals [51,52] may stimulate the interest of potential consumers [53], especially in terms of a self-care decision. In this case, such decisions need to be evidence-based.

We conducted a randomized controlled trial to investigate the overall impact of quercetin and standard-of-care anti-hyperglycemic agents on cardiometabolic and psychosocial health. This study explored the potential of this combination as a plausible integrative strategy for diabetes care. The primary goal of our study included the assessment of the effects of quercetin phytotherapy on selected bio-clinical markers, such as glycated hemoglobin, blood pressure, lipids, and lung function parameters, whereas as the secondary objective, this study assessed quercetin’s impact on psychosocial and life habit outcomes (night sleep duration, anxiety, and quality of life).

## 2. Materials and Methods

### 2.1. Phytochemical Supplement Specifications

This study utilized a commercially available, standardized quercetin dihydrate supplement containing a 500 mg dosage per serving (batch code F160996). The supplement is registered with the Greek National Organization for Medicines (disclosure no. 75608/20.11.2008). Prior independent analysis verified its quercetin content to be within the manufacturer’s specification [54].

### 2.2. Study Conduct: Ethical Approval and Research Compliance

This study was prospectively registered with ISRCTN [55] in November 2022. It was approved by the University of Crete Research Ethics Committee (104/20-08-2021) and the 7th Health District of Heraklion (6380-14/02/2022). This randomized controlled trial was designed in accordance with the Declaration of Helsinki [56] and ICH E6(R2) Good Clinical Practice guidelines [57]. This trial diligently complied with the World Health Organization and European Medicines Agency clinical practice guidelines [58,59], as well as with European Regulation 536/2014 [60]. A parallel-group structure was employed, and reporting adhered to CONSORT guidelines [61].

### 2.3. Study Design

This study was a prospective, two-arm, single-center, parallel-group randomized controlled trial (RCT) to investigate the efficacy (clinical usefulness and feasibility) of quercetin as complementary support in patients with T2DM receiving usual care with non-insulin medications. The main study was performed in a primary healthcare (PHC) facility, the 4th Local Health Unit of Heraklion (4th TOMY), Crete (patients’ enrollment: February–May 2023). After a detailed explanation of the procedures of this study and of their right to withdraw at any point, all participants voluntarily granted written informed consent. 

Participant inclusion was determined by age over 50 years old during preliminary assessment, a clinical T2DM diagnosis confirmed by medical history, and current non-insulin diabetic medication treatment. Exclusion criteria addressed high-risk groups or individuals as detailed in the published interim research initiative [42].

The review of medical records identified 324 potential participants meeting the inclusion criteria. A detailed assessment of those suitable for inclusion followed, and 163 were contacted for eligibility. Ten of them reported not meeting the inclusion criteria and fifty-three declined active involvement. We ultimately enrolled 100 patients, following the allocation plan generated by an independent researcher after randomization to minimize selection bias. 

Stratified randomization by age and sex (1:1 ratio) assigned participants to control (CTR, n = 50) or intervention (INT, n = 50) groups. The CTR group continued their standard care, while the INT group received the manufacturer-recommended daily quercetin dihydrate supplement dosage (500 mg) for 12 weeks, followed by an 8-week washout and a consecutive 12-week supplementation period (total 32 weeks) as supportive treatment (in combination with their usual therapy). Standardized guidance was provided consistently to both groups by a trainer researcher. All patients were instructed to take the supplement after meals, as per container instructions.

Phone monitoring by a designated researcher ensured patient compliance. Strategies to enhance the scheduled supplement intake adherence included clear instructions, staggered provision of supplement containers, pill counts, and treatment reminders. Reasons for non-adherence, incomplete treatment, or withdrawal were documented.

**Figure 1:**
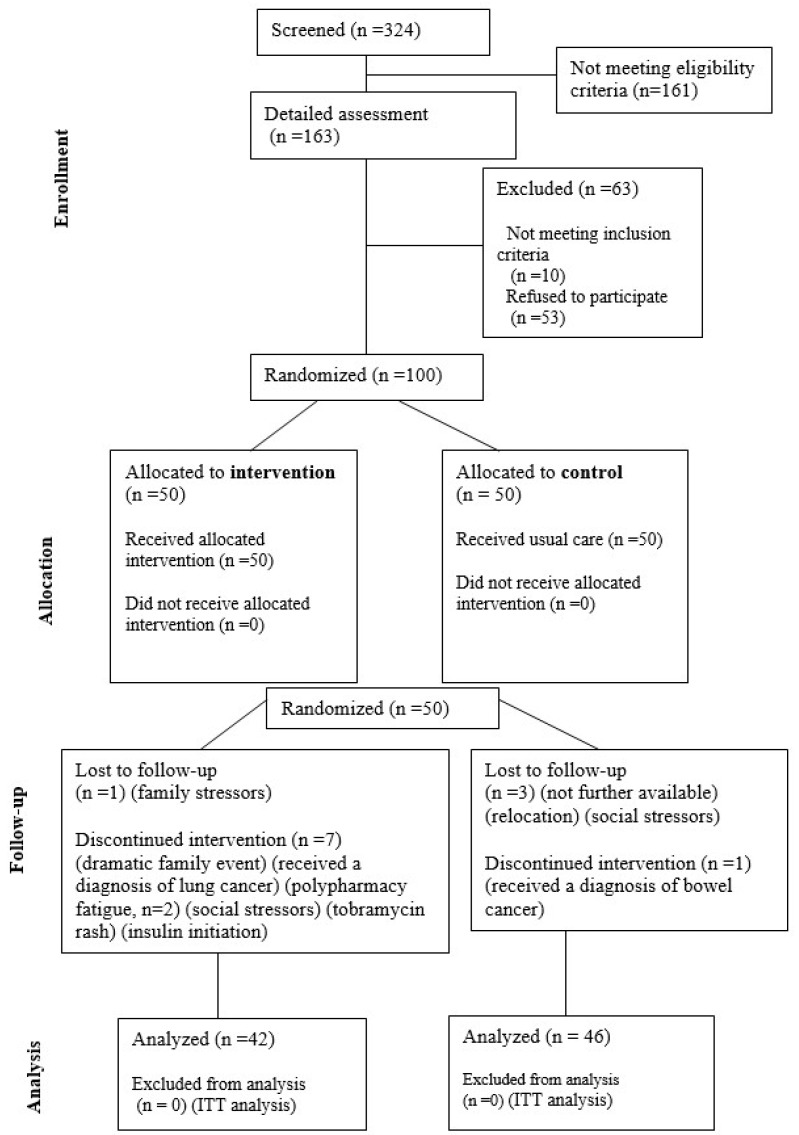
Flow diagram of this study as per CONSORT guidelines.

Participant progression through the randomized trial is illustrated in Figure 1 (CONSORT diagram), summarizing the reasons for loss of follow-up.

Scheduled follow-up visits occurred in person. Specifically, the first visit took place at baseline, with a subsequent intermediate encounter for a randomized subset of patients at 3 months (June–July 2023) to investigate initial efficacy signs and potential clinical safety concerns [42], and at the endpoint (September 2023–January 2024). The intermediate 3-month follow-up was conducted at participants’ homes, whereas the other two occurred at the study site (4th TOMY).

Study participants underwent a comprehensive health assessment. This included a researcher-administered health information survey, biometric (PiKo-6 spirometry, BMI, waist circumference, blood pressure) and clinical profiling (smoking and drinking habits, multimorbidity, polypharmacy, vaccination, years since T2DM diagnosis, age, night-time sleep, moderate-intensity activities), and laboratory blood tests to assess hematological parameters (complete blood count, CBC), inflammation (c-reactive protein, CRP), glucose metabolism (HbA1c), lipid status (total cholesterol, low density lipoprotein, triglycerides), renal function (creatinine, serum urea), reticulocyte count, and levels of DHEAS and 25-OH-vitamin D. Additionally, we used the 36-item Short Form Health Survey (SF-36) [62,63,64] and Short Anxiety Screening Test (SAST-10) [65,66] to collect QoL and anxiety data, respectively. 

Patients’ morning blood samples were obtained at baseline and study termination following a standardized overnight fasting protocol of at least 8 h. Following phlebotomy, they were aliquoted into separate tubes and immediately used for analyses conducted by healthcare analytics providers operating under strict, internationally aligned quality standards. For preservation, the samples were de-identified and immediately frozen at −20 °C to eventually serve posterior study purposes for consenting patients. Analytics provider staff were blinded to treatment assignments.

Widely accepted measurement techniques and validated tools were used to assess health parameters at follow-ups. These were explicitly detailed in the intermediary report of this study [42]. 

### 2.4. Clinical Safety and Tolerability

To assess tolerability and safety throughout this study, participants were interviewed regarding any side effects or adverse events experienced during routine telephone calls and follow-up visits. Serum urea and blood creatinine levels were measured at baseline and study termination to evaluate changes in renal function. Additionally, as all patients were recruited from the registered population of the involved PHC setting (4th TOMY), their assigned primary care physicians actively monitored their health status.

### 2.5. Sample Size Estimation

A power analysis was conducted with G*Power 3.1.9.7 [67,68] to estimate the required sample size. To reach a 0.8 power, significance level of α = 0.05, and a 0.6 effect size, a total sample size of 90 subjects (divided into two groups) was calculated. Taking into account a potential 10% attrition rate, the final target sample size was increased to 100 subjects. This sample size aligns with the methodology used in similar randomized clinical trials [69,70,71] and exceeds sample sizes used in comparable studies [36,72].

### 2.6. Statistical Methods

Statistical analyses were conducted through the Statistical Package for the Social Sciences (SPSS) software (IBM Corp. Released 2019, IBM SPSS Statistics for Windows, version 25.0, Armonk, NY, USA: IBM Corp.). We assessed data normality using Blom’s method (Q-Q plot). Data were presented as measures of central tendency and dispersion or frequency distributions, using percentage changes (Δ-%). We analyzed parametric data by independent-samples *t*-tests for between-group comparisons. Δ-changes were calculated, and statistical significance was indicated by a *p*-value < 0.05. Effect sizes were presented using Cohen’s d. 

## 3. Results

A total of 88 participants (48 men and 40 women) completed the 32-week follow-up period (final endpoint). Attrition included 12 participants, 8 in the INT and 4 in the CTR group. An intention-to-treat (ITT analysis) was employed, including 42 patients from the intervention and 46 from the control group.

Rigorous measures were taken to enhance treatment adherence, as mentioned earlier. Overall, the scheduled nutraceutical intake within the treatment arm demonstrated comparability and the patients conformed to protocol requirements.

The age range of the participants was 50 to 79 years. Participants’ mean age was 66.9 ± 7.7 years (INT) and 68.7 ± 6.6 years (CTR). The prevalence of current smoking and alcohol consumption was similar, with 14 and 16 current smokers in the INT and CTR groups, respectively, and 29 and 30 current alcohol drinkers.

Multimorbidity was observed in 33 patients within the INT group and 31 within the CTR group. For the most frequent chronic conditions (viz., hypertension, dyslipidemia, chronic obstructive pulmonary disease (COPD), asthma, asthma–COPD overlap syndrome (ACOS), depression, and anxiety), there was no statistically significant difference between the control and intervention groups (*p* > 0.05). All patients in both groups were treated with oral anti-diabetic pills (i.e., metformin, DPP-4 and SGLT-2 inhibitors, sulfonylurea, or their combinations without statistically significant differences between groups; *p* > 0.05), with eight in each group also receiving GLP-1 receptor agonists (INT: 34/8 vs. CON: 38/8; *p* = 0.840). Polypharmacy was also comparable, with 29 subjects in the INT group and 32 subjects in the CTR group taking multiple medications. Vaccination records showed high rates across both groups for influenza (INT: 35, CTR: 40), pneumococcal (INT: 30, CTR: 32), and shingles vaccines (INT: 14, CTR: 20). Notably, nearly all participants had received the COVID-19 vaccine (INT: 41, CTR: 45).

These are further displayed in Table 1. 

Notably, no adverse side effects associated with quercetin supplementation were reported by the patients. One patient experienced allergy symptoms due to tobramycin, non-related to this study, and another reported mild, transient gastro-intestinal symptoms in week 31, the latter being subsequently reported at the endpoint meeting (32 weeks). Additionally, no concerns related to the active arm of this study were disclosed by the general practitioners (GPs) of the involved PHC setting. Furthermore, all measured laboratory markers remained within physiological norms and no clinically significant changes were discerned throughout the study period, as depicted in Table 2 and Table 3. The absence of side effect incidences further supports a potentially favorable tolerability and advantageous adverse event profile of this phytochemical.

**Figure 2:**
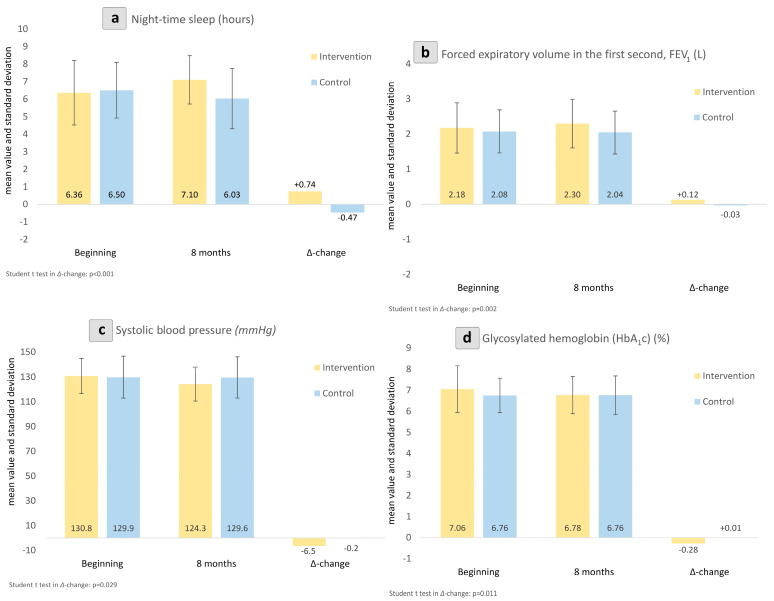
Between-group changes in night-time sleep (**a**), forced expiratory volume in the first second (FEV_1_) (**b**), systolic blood pressure (**c**), and glycated hemoglobin (HbA1c) (**d**). Intra-group changes shown as Δ-change from beginning to endpoint of this study.

The intervention and control groups were well composed at baseline with respect to their anthropometric, demographic and clinical characteristics, as presented in Table 1, Table 2 and Table 3. In our study, some nationality variation does not reflect any race diversity, since all study participants shared a West Eurasian ancestry. At study entry, the groups indicated similarities regarding night-time sleep, body mass index (BMI), waist circumference, forced expiratory volume in the first second (FEV_1_), forced expiratory volume in the sixth second (FEV_6_), blood pressure (BP), and SF-36 and SAST-10 scores. Additionally, the mean duration since the diagnosis of T2DM was comparable across all participants. Table 4 displays the changes in the examined health parameters during the 32-week-long study period in the control and intervention groups. Specifically, pre/post comparisons within the quercetin group revealed a statistically significant increase in mean night-time sleep compared to the control group (+0.74 vs. −0.47 h; *p* < 0.001). Similarly, a statistically significant increase was observed in FEV_1_ (0.12, −0.03 L; *p* = 0.002) in comparison to the control group. Systolic BP was significantly ameliorated in the intervention arm in contrast to the control group (−6.55, −0.24 mmHg; *p* = 0.029). These are further visualized in Figure 2.

**Figure 3:**
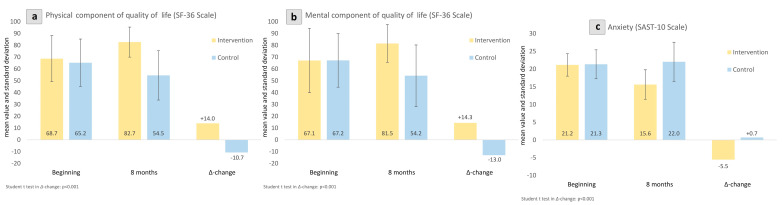
Between-group changes in physical and mental component of quality of life assessed by SF-36 (**a**,**b**) and anxiety levels examined by SAST-10 (**c**). Intra-group changes visualized as Δ-change from the beginning to the endpoint of this study.

**Figure 4:**
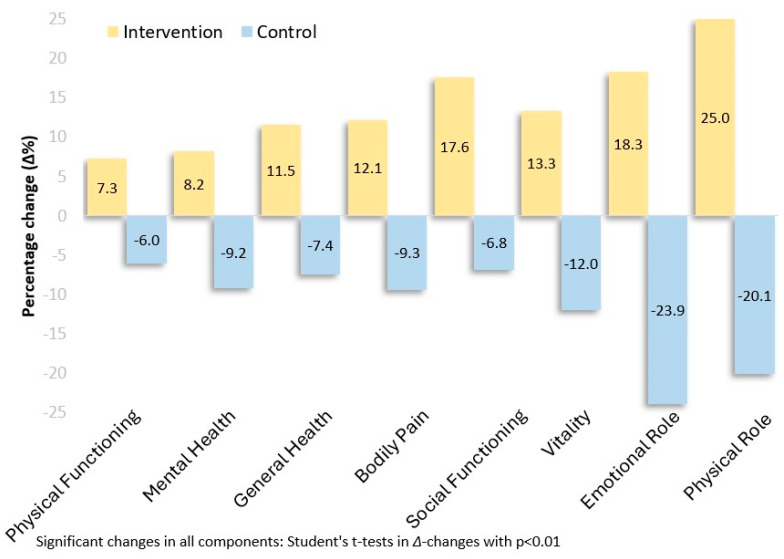
Percentage change (Δ%) in the 8 components/subscales of the SF-36 Quality of Life Scale from the beginning to eight months of this study among 88 patients with T2DM randomized to intervention (n = 42) and control (n = 46) groups.

Both the physical (13.99, −10.71; *p* < 0.001) and mental component (14.33, −12.97; *p* < 0.001) of QoL (SF-36 Scale) were found to be significantly improved in the intervention group. Anxiety levels assessed by SAST-10 were favorably influenced in the intervention (−5.55, 0.70; *p* < 0.001) compared to the control group. These results are delineated in Figure 3 and Figure 4.

Also, non-significant, post-supplementation alterations were surveyed. In particular, moderate-intensity activities in the last 7 days for the intervention group changed minimally, as affirmed by Δ-change (INT: 0.56, CTR: 0.79 days; *p* = 0.586). Additionally, BMI presented a minor decrease in the intervention group (INT: −0.47, CTR: −0.30 kg/m^2^; *p* = 0.463) and, correspondingly, so did waist circumference (INT: −0.88, CTR: −0.46 cm; *p* = 0.704). Likewise, diastolic blood pressure displayed a minor alteration (INT: 1.74, CTR: 1.11 mmHg; *p* = 0.741). Interestingly, forced expiratory volume in 6 s (FEV_6_) revealed near-significance trends in the quercetin group (INT: 0.27, CTR: 0.10 L; *p* = 0.055). 

The cholesterol ratio (total cholesterol/high-density lipoprotein) showed minimal decline from the intra-group comparison of the active arm. This finding reached marginal statistical insignificance (*p* = 0.061) with a moderate effect size. In contrast, total cholesterol, triglycerides and low-density lipoprotein (LDL) were not significantly affected in the INT group. 

Self-descriptive insights derived from interview analysis revealed a range of improvements in the active arm. Physical advancements included increased mobility (n = 2), reduced pain (n = 3), improved weight management (n = 1) and decreased appetite (n = 1). Additionally, two patients reported better blood pressure measurements. Other improvements included reduced arthritic symptoms (n = 2), enhanced mood (n = 6), and reduced fatigue (n = 2). Further benefits comprised increased energy levels (n = 6), improved sleep (n = 1), enhanced daily self-management (n = 3), and improved intestinal motility (n = 1). One patient noted a scaled-down diabetes treatment plan after medical consultation, while four reported improved glucose regulation. Finally, six patients expressed increased interest and follow-through, with two motivated to recommend quercetin to family members. 

## 4. Discussion

The current study investigated the potential of quercetin to be used as added nutritherapy for type 2 diabetes mellitus (T2DM). Our findings underscore a significant positive influence of 500 mg daily quercetin dosage as supplement intake on night-time sleep, PiKo-6 readings, blood pressure, glycated hemoglobin, anxiety, and QoL in a T2DM cohort. However, no significant changes were observed in moderate-intensity activities in the last 7 days, BMI and correspondingly waist circumference, diastolic blood pressure, and FEV_6_ levels (*p* > 0.05). No clinically negative issues were observed in the treatment arm during the study period. Our previous midpoint analysis at 8 weeks of oral administration demonstrated trends consistent with those observed at endpoint [42].

Importantly, our study substantiates the promising anti-hyperglycemic action of quercetin by assessing HbA1c, a valuable marker to assess overall glycemic control, with limited investigation in humans. To the best of our knowledge, only two human studies have been conducted to investigate this effect to date. Mazloom and colleagues (2014) in a single-blinded RCT administered 250 mg of quercetin to 47 patients with T2DM for 8 weeks but did not observe statistically significant changes compared to placebo [73]. Similarly, in an RCT by Brüll and colleagues (2017), 162 mg/day onion-skin-extracted quercetin was provided for 6 weeks to overweight-to-obese patients with pre- and stage 1 hypertension and no inter-group statistical significance was detected [74]. 

Antioxidant intake may improve blood sugar levels in diabetes by countering oxidative stress. Potential mechanisms include direct scavenging of reactive oxygen species (ROS) to protect insulin-producing cells and insulin signaling, upregulation of antioxidant enzymes, and reduction in advanced glycation end products (AGEs) that promote inflammation [75]. Moreover, flavonoids show potential in regulating blood sugar levels by acting upon multiple pathways. They may enhance glucose uptake into cells by influencing glucose transporters, reduce glucose production in the liver by targeting key enzymes, and improve insulin sensitivity through modulation of signaling pathways like AMPK and PPAR [76]. Likewise, a systematic review and meta-analysis of RCTs revealed statistically significant improvements in lipid and glucose outcomes following flavonoid consumption [77]. Our results follow the same direction as those exerted by another nutraceutical, berberine [78].

Moreover, systolic BP exhibited a clinically significant decrease, whereas diastolic BP demonstrated a minimal change in the supplemented cohort. These findings converge with a systematic review and meta-analysis by Elfaituri and colleagues, which supports that quercetin supplementation may significantly reduce systolic blood pressure in both hypertensive and normotensive individuals. However, the authors agree that its effect on diastolic blood pressure appears negligible [79]. This flavonoid may lower blood pressure, potentially through contributing to cytosolic elevation of chloride [Cl^−^] in renal epithelial cells, which in turn decreases epithelial sodium channel (ENaC) gene expression by reducing renal sodium [Na^+^] reabsorption [80]. It may also induce vasorelaxation [80].

Furthermore, pairwise comparisons using Student’s *t*-tests within the quercetin-supplemented group detected a statistically significant increase in FEV_1_, with a moderate effect size and with no significant change in regard to FEV_6_. Our study provides further insights into a recently explored effect of quercetin supplementation, representing only the second human study in this field. Lung function benefits following senolytic treatment using a combination of quercetin and dasanitib were only recently assessed in humans in a single, two-center, 3-week pilot study involving merely 14 patients with Idiopathic Pulmonary Fibrosis (IPF). The authors concluded that senolytics may modulate inflammation and tissue remodeling in IPF, as evidenced by reduced MCP-1 and matrix remodeling proteins, while concurrently improving physical function in IPF, albeit not captured by conventional spirometry [46]. A possible improvement in lung function may stem from a strengthening of the diaphragm. Indeed, a muscle-strengthening effect may be a more plausible explanation for the described study findings. This possibility is consistent with previous research showing a linkage between oxidative stress and weakened diaphragm function [81]. A clinical trial in COPD patients also revealed that quercetin might alleviate lung inflammation [82]. Specifically, this anti-inflammatory action may present multiple benefits for lung function, such as reduced cellular infiltration, lowered cytokine levels, decreased oxidative damage, and other protective mechanisms [83]. Our findings also coincide with prior animal studies where quercetin was shown to have beneficial effects on lung function [83,84]. 

Our findings suggest that quercetin may significantly enhance night-time sleep duration in the population studied. This observation assumes additional significance when considered alongside research indicating that chronic sleep deprivation may contribute to accelerated cellular aging [85] and negatively affects health outcomes [86]. Our findings align with animal model research suggesting a potential modulation of GABA(A) receptors by quercetin and its metabolite, quercetin-3-O-glucuronide [87,88], and an RCT including 78 chronically fatigued patients which found improved Pittsburgh Sleep Quality Index scores among the treatment group [89]. Nonetheless, a prior randomized, double-blind controlled trial among military trainees found no significant effect of quercetin on sleep quality or fatigue [90]. Contrarily, our outcomes correspond to the sleep-promoting effects documented for *Bacopa monnieri* extract in individuals experiencing sleep deprivation [91]. 

Until now, anxiety mitigation by quercetin was unexplored in human studies. The anxiolytic effect captured by this study parallels that of *Nepeta menthoides* supplementation in patients with depressive symptoms [92]. Hyperglycemia-induced oxidative stress may play a role in the pathophysiology of anxiety disorders. Elevated oxidative stress can potentially lead to the overexpression of pro-inflammatory cytokines, resulting in neuroinflammation and subsequent neurodegeneration [48], implicating complex mechanisms underlying the protective function of flavonoids [93].

Research suggests that gut bacteria play a critical role in transforming this phytochemical compound into bioactive forms [94]. Interestingly, oral administration of quercetin produced anxiolytic effects in male mice, while abdominal injection did not, implying a strong dependence on the route of administration [95]. Moreover, the degree of hydroxylation at specific ring positions of the flavonoid appears to influence its activity [95]. Also of note, the metabolite of quercetin, 3-4-dihydroxyphenylacetic acid, exhibits enhanced antiplatelet activity compared to the parent compound [96]. Crucially, the anxiolytic effects of orally administered quercetin in a *Mus musculus* model were eliminated after antibiotic treatment, highlighting the essential role of gut microbiota in transforming quercetin into its biologically active metabolites [96]. Our findings align with previous studies in murine models which demonstrate the anxiolytic effects of quercetin, potentially mediated by a reduction in hippocampal oxidative stress [97,98,99,100].

This study’s results show that various aspects of QoL may be positively affected by quercetin intake in patients with T2DM, in conjunction with favorably influenced anxiety levels. Existing evidence suggests a negative association between aging and QoL [101,102]. In fact, emerging research denotes a relation between anxiety disorders and accelerated signs of neurodegeneration in the elderly [103]. Furthermore, higher anxiety levels have been linked to poorer QoL in older populations [103]. Notably, anxiety related to aging itself has been implicated in a decline in QoL [104]. The importance of prioritizing QoL enhancements within diabetes management protocols is underscored.

QoL is frequently affected in those living with T2DM [105]. Although achieving full disease control remains unattainable, mitigation of further QoL deterioration is paramount for integrative diabetes care [106]. While previous research on the effect of quercetin on QoL is limited, promising findings exist. Dehghani et al. (2021) reported improved QoL with quercetin supplementation in post-myocardial infarction patients, particularly regarding feelings of uncertainty [70]. Our interim observation in the three-month quercetin intake interval in regard to its benefits on QoL remained unchanged and significantly robust [42]. Our study hints at broader QoL improvements, extending those observed in a similarly dosed RCT of a shorter duration and a different population [89], yet are harmonized with the influence of a *Mentha pulegium* extract on patients suffering from functional dyspepsia [107]. Further investigation is warranted to unravel the potential of quercetin in enhancing these psychosocial aspects.

In this intervention, BMI exhibited a minor decrease compared to the control group. This aligns with prior research, since a three-arm, parallel-group, 12-week RCT in socially burdened adults found no significant BMI differences between 500 mg or 1000 mg quercetin supplementation dosages [108]. Correspondingly, evidence from a recent systematic review and meta-analysis of RCTs shows that BMI is unaffected by quercetin administration [109]. Nevertheless, a more recent, 12-week RCT investigating the advantages of quercetin for metabolic syndrome revealed a significant decrease in BMI among supplemented participants, despite a lower dosage (240 mg) [110]. 

Waist circumference demonstrated a minimal, non-significant decrease in the quercetin group, despite the proposed, not fully understood mechanisms of action of quercetin on waist circumference. These involve anti-inflammatory action on adipose tissue and modulation of the AMPK, ERK, and JNK signaling pathways [111]. Our findings mirror those of a recent systematic review and meta-analysis of RCTs [109] yet contrast with another crossover RCT including 49 men grouped by APOE genotype [111]. 

The cholesterol ratio yielded a minor decline in the treatment arm. Conversely, total cholesterol, triglycerides, and low-density lipoprotein (LDL) showed no significant alterations in the INT group. This further corroborates earlier RCT studies analyzed in a systematic review and meta-analysis by Sahebkar et al., who assessed the impact of quercetin supplementation on circulating lipid levels. The authors observed a statistically significant reduction in plasma triglyceride concentrations at doses of 500 mg/day or higher and an administration duration of 4 or more weeks. Notably, quercetin supplementation did not exert a statistically significant influence on LDL or high-density lipoprotein (HDL) levels [112]. 

A recent mouse model suggests that quercetin promotes the expression of scavenger receptor class B type I (SR-BI), a protein crucial for the selective upregulation and clearance of HDL-cholesterol. Quercetin may achieve this effect through the activation of the PPARγ/LXRα pathway [113]. Given the role of HDL in facilitating cholesterol clearance [114], the potential of quercetin to modulate HDL function suggests it may favorably influence the cholesterol ratio. Nevertheless, the literature is inconclusive since another recent systematic review and meta-analysis found that overall lipid and glucose profiles remained largely unaffected by quercetin supplementation. However, in trials using parallel designs, quercetin consumption for at least 8 weeks produced notable changes in both HDL and triglyceride levels [115]. This highlights the necessity to further investigate dosage, duration, and study design as key factors influencing the impact of quercetin on lipid metabolism.

A statistically insignificant increase in platelet count in the intervention group (Δ%-change INT: 4.3, CTR: −0.9; *p* = 0.171) could reflect quercetin’s potential modulation of cellular senescence pathways, a major factor in age-related physiological changes. Age-related declines in bone marrow along with increased apoptosis indicate a potential mechanism of reduced platelet production with advancing age [116]. Studies suggest this decline in platelet production and subsequent decrease in platelet count [116,117] are positively correlated with increasing age. 

Similar minimal non-significant trends were observed in the reticulocyte fraction (INT: 9.3, CTR: −15.2; *p* = 0.277). Reticulocytes are immature red blood cells which mature into functional red blood cells, replacing older red blood cells [118]. Stimulatory stressors can increase the proportion of immature reticulocytes in the bloodstream and lengthen their maturation time [118]. This might be particularly relevant in the context of aging, where reduced bone marrow activity could act as a stressor, potentially impacting reticulocyte distribution. Significant decreases in reticulocyte count with increasing age have been reported in the literature [119,120].

Given the potential of quercetin for therapeutic applications, understanding its pharmacokinetics is crucial. The molecule and its metabolites possess a notably long half-life, ranging from 11 to 28 h, suggesting that repeated supplementation could lead to a substantial increase in plasma levels [121]. This extended half-life underscores the importance of careful dosage considerations and monitoring during clinical trials involving quercetin. Moreover, typical dietary quercetin consumption, generally within the range of 6 to 18 mg/day, proves insufficient to induce a significant plasma response. To attain desired therapeutic outcomes, supplementation surpassing 50 mg of quercetin aglycone or its equivalents is frequently necessary [121]. Thus, duration and dosage trialing is crucial to establish the optimal regimen.

What also emerges as an intriguing insight is the intricate interplay between the observed beneficial outcomes. Indeed, the literature affirms that sleep deprivation is linked to elevated HbA1c levels [122,123], and restrained spirometry parameters have been associated with higher HbA1c levels, indicating poor glycemic control [124]. Moreover, psychological distress has been identified as a factor negatively affecting HbA1c fraction [125,126,127]. Importantly, insufficient glycemic control has similarly been proven to be related to hypertension incidence [128,129], with evidence illuminating their reciprocal interaction [130]. 

Social prosperity offers enhanced standards of living enabled by technological progress; however, this coincides with an ominous increase in diabetes cases. Consequently, diabetes emerges as a substantial health concern of the 21st century [7].

Our investigation demonstrates that quercetin offers potential benefits within a primary healthcare context, and specifically for patients with T2DM. Its anxiolytic potential suggests an alternative or additional option enhancing patient well-being. Moreover, favorable outcomes point towards the utility of quercetin as a component of comprehensive, patient-centered supportive care in diabetes management. These findings necessitate additional research to explore the long-term efficacy and translational potential of quercetin within primary care settings.

Through placing emphasis on a PHC environment, our study ensures the inclusion of a patient group representative of real-world clinical practice. We believe this methodology strengthens potential insights into the value of quercetin as a supplement regimen for patients managing T2DM, with a clearly recommended, firmed compliance with international guidelines and established usual treatments.

### Study Strengths and Limitations

This study implemented a two-arm, randomized controlled trial design to assess the pleiotropic benefits of quercetin for patients with T2DM. Although random allocation established similar baseline groups, the strengths of this study must be weighed against its limitations. 

This RCT translates promising findings from animal models into the human context, being the first exploratory effort to assess the effects of quercetin on anxiety. While the present study demonstrated substantial benefits of quercetin on anxiety, the potential for more pronounced effects with higher dosages warrants further investigation. Our preliminary analysis yielded strong trends, offering initial support for a potential relationship [42]. Notably, this study contributes to the limited human research examining the anti-hyperglycemic effect of quercetin by evaluation of HbA1c. To the best of our knowledge, only one study has examined lung function aspects concomitant to quercetin administration [46] and few have assessed the role of quercetin in QoL amelioration [70,89].

Our study focused on the combination of quercetin supplementation with usual pharmacotherapy as adjunct supportive care. It included a control group receiving only usual care, allowing for comparisons. Moreover, our study, while investigating several laboratory markers, vital signs, and psychosocial, life habit, and cardiometabolic measures, did not assess other glycemic outcome measures in T2DM, such as the impact on insulin sensitivity.

Our study examined both objective and subjective outcome variables, with the latter being prone to confounding patient expectations, attributable to an open-label protocol. This applies especially to the observation of a strikingly beneficial effect of treatment on measures of quality of life. We also need to stress that the impact on quality of life was not a primary goal of this study. The link between quality-of-life trends and changes in bio-clinical features suggests that quality-of-life benefits may, in part, be driven by an ‘active-treatment’ effect. Thus, due to these uncertainties, the quality-of-life trends might usefully be viewed as providing ‘proof-of-concept’ and justifying a definitive placebo-controlled trial, in terms of double-blindness. 

Furthermore, this study did not measure quercetin metabolites in plasma or urine, limiting conclusions on its bioavailability, which could add supplementary value when considering its potential antioxidant or anti-inflammatory function.

Another limitation of this study was the limited capacity to manage the participants’ lifestyle. However, the provision of uniform guidance may have partially mitigated this effect in both groups. 

Future studies should incorporate larger and multidisciplinary designs, double-blind trialing, different dosages, and scheduled duration, mechanistic explorations, multiple sites and more diverse participant groups to further bolster the robustness of the outcomes. Notwithstanding this, the sample size of this study closely adhered to the initial study design, and the loss to follow-up, even if within typical range, highlights the importance of further research to definitively confirm our findings. It also produced some further research confidence that randomized controlled trialing is feasible in a primary care environment with limited resources in order to improve capacity and patient-tailored care [131,132].

Finally, focusing specifically on patients with T2DM, who often exhibit accelerated aging [85], this study nurtures research expectations of detecting potential benefits to healthier individuals even within the same age group, as future implications through similar research initiatives.

## 5. Conclusions

This study was designed to investigate potential health benefits associated with quercetin administration in patients with T2DM. An anti-hyperglycemic (HbA1c), normotensive (systolic BP), and increased one-second expiratory airflow (FEV_1_) potential emerged and warrants further investigation, while no statistically significant effects on diastolic blood pressure, BMI, and, correspondingly, waist circumference were observed. Our findings also posit that quercetin supplementation (500 mg daily) may increase night-time sleep duration and positively impact anxiety burden and quality of life (QoL). Additional investigations with varying quercetin dosages and durations are needed to establish its therapeutic potential and optimal parameters for health improvement and to shed light on long-term effectiveness and safety of quercetin. This study underscores the possibility of incorporating quercetin into diabetes research within a PHC environment. The results indicate that quercetin has the potential to provide an integrative, patient-focused approach as supportive care for patients with T2DM, alongside established pharmacological treatment.

## Figures and Tables

**Table 1:** Descriptive characteristics of 88 patients with type II diabetes (T2DM) randomized to intervention and control groups.

		Groups	
		Intervention (n = 42)	Control (n = 46)
		**n (%)**	
**Gender**	male	24 (57.1)	24 (52.2)
	female	18 (42.9)	22 (47.8)
**Age,** years	mean ± stand. dev.	66.9 ± 7.7	68.7 ± 6.6
**Nationality**	Greek	38 (90.5) *	46 (100.0)
**Current smoker**	yes	14 (33.3)	16 (34.8)
**Current drinker**	yes	29 (69.0)	30 (65.2)
**Multimorbidity**	3+ chronic conditions	33 (78.6)	31 (67.4)
**Polypharmacy**	4+ medications	29 (69.0)	32 (69.6)
**Vaccination**	influenza	35 (83.3)	40 (87.0)
	pneumococcal	30 (71.4)	32 (69.6)
	shingles (herpes zoster)	14 (33.3)	20 (43.5)
	COVID-19	41 (97.6)	45 (100.0)
**Years diagnosed with T2DM**	mean ± stand. dev.	11.1 ± 6.9	10.2 ± 6.6

Chi-square (χ^2^) and Student’s *t*-tests: * *p* < 0.05.

**Table 2:** Levels of and changes in hematology measurements of the 88 patients with T2DM randomized to intervention (n = 42) and control (n = 46) groups, from the beginning to eight months of this study.

		Groups			Cohen’s *d* Effect Size
		Intervention	Control	*p*-Value	
		Mean ± Stand. Dev.			
**Lymphocytes (LYMPH)** (%)	beginning	31.79 ± 6.20	31.65 ± 6.87		
	8 months	31.22 ± 8.75	30.98 ± 7.85		
	**Δ-change**	**−0.57**	**−0.67**	0.941	0.02
	Δ%-change	−1.8	−2.1		
**Monocytes (MONO)** (%)	beginning	5.92 ± 1.98	6.35 ± 2.07		
	8 months	6.65 ± 2.47	6.40 ± 1.71		
	**Δ-change**	**0.72**	**0.05**	0.186	0.29
	Δ%-change	12.2	0.8		
**Neutrophils (NEUT)** (%)	beginning	58.83 ± 7.22	58.84 ± 7.18		
	8 months	58.84 ± 9.71	59.00 ± 8.52		
	**Δ-change**	**0.02**	**0.15**	0.931	0.02
	Δ%-change	0.0	0.3		
**Hemoglobin (HGB)** (g/dL)	beginning	15.69 ± 4.49	13.75 ± 1.44		
	8 months	14.42 ± 1.38	13.75 ± 1.43		
	**Δ-change**	**−1.26**	**0.00**	0.092	0.37
	Δ%-change	−8.1	0.0		
**Platelet count (PLT)** × 10^3^/mm^3^	beginning	227.0 ± 53.1	243.0 ± 54.1		
	8 months	236.9 ± 53.9	240.9 ± 65.0		
	**Δ-change**	**9.8**	**−2.1**	0.171	0.30
	Δ%-change	4.3	−0.9		
**White blood cells (WBCs)** × 10^3^/μL	beginning	7.87 ± 2.12	7.48 ± 2.03		
	8 months	7.85 ± 2.41	7.47 ± 1.94		
	**Δ-change**	**−0.01**	**−0.01**	0.987	0.00
	Δ%-change	−0.2	−0.1		
**Reticulocytes (RTCs)** %	beginning	1.36 ± 0.50	1.57 ± 2.03		
	8 months	1.45 ± 0.51	1.33 ± 0.42		
	**Δ-change**	**0.13**	**−0.24**	0.277	0.24
	Δ%-change	9.3	−15.2		

Student’s *t*-tests in Δ-changes (in bold).

**Table 3:** Levels of and changes in biochemical measurements of the 88 patients with T2DM randomized to intervention (n = 42) and control (n = 46) groups, from the beginning to eight months of this study.

		Groups			Cohen’s *d* Effect Size
		Intervention	Control	*p*-Value	
		Mean ± Stand. Dev.			
**Serum urea** (mg/dL)	beginning	34.95 ± 9.62	37.37 ± 11.80		
	8 months	35.69 ± 7.90	36.66 ± 10.81		
	**Δ-change**	**0.74**	**−0.72**	0.478	0.15
	Δ%-change	2.1	−1.9		
**Blood creatinine** (mg/dL)	beginning	0.80 ± 0.22	0.83 ± 0.25		
	8 months	0.82 ± 0.25	0.87 ± 0.27		
	**Δ-change**	**0.02**	**0.04**	0.461	0.16
	Δ%-change	1.9	4.4		
**Total cholesterol** (mg/dL)	beginning	153.8 ± 35.4	162.6 ± 37.4		
	8 months	159.1 ± 36.1	162.4 ± 44.1		
	**Δ-change**	**5.3**	**−0.2**	0.417	0.18
	Δ%-change	3.5	−0.1		
**Triglycerides** (mg/dL)	beginning	146.9 ± 85.3	136.7 ± 59.0		
	8 months	154.4 ± 80.5	146.3 ± 62.5		
	**Δ-change**	**7.5**	**9.6**	0.825	0.05
	Δ%-change	5.1	7.0		
**Low-density lipoprotein** (LDL) (mg/dL)	beginning	79.8 ± 33.7	86.0 ± 32.6		
	8 months	85.4 ± 34.4	87.3 ± 33.8		
	**Δ-change**	**5.6**	**1.3**	0.509	0.14
	Δ%-change	7.1	1.5		
**C-reactive protein (CRP)** (mg/dL)	beginning	0.97 ± 2.80	0.76 ± 1.67		
	8 months	0.78 ± 1.58	0.62 ± 1.11		
	**Δ-change**	**−0.19**	**−0.14**	0.892	0.03
	Δ%-change	−19.7	−18.2		
**Glycated hemoglobin (HbA_1_c)** (%)	beginning	7.06 ± 1.11	6.76 ± 0.82		
	8 months	6.78 ± 0.89	6.76 ± 0.92		
	**Δ-change**	**−0.28**	**0.01**	0.011	0.56
	Δ%-change	−4.0	0.1		
**25-hydroxy vitamin D** (ng/mL)	beginning	33.8 ± 15.8	30.4 ± 16.3		
	8 months	28.6 ± 11.6	29.0 ± 9.7		
	**Δ-change**	**−5.2**	**−1.4**	0.248	0.25
	Δ%-change	−15.4	−4.5		
**DHEA sulfate (DHEAS)** (μg/mL)	beginning	0.91 ± 0.57	0.87 ± 0.64		
	8 months	0.81 ± 0.48	0.94 ± 0.89		
	**Δ-change**	**−0.10**	**0.07**	0.078	0.39
	Δ%-change	−11.2	7.5		
**Cholesterol ratio**	beginning	3.93 ± 1.96	3.35 ± 0.92		
	8 months	3.85 ± 1.44	4.09 ± 2.10		
	**Δ-change**	**−0.08**	**0.74**	0.061	0.41
	Δ%-change	−1.9	22.0		

Student’s *t*-tests in Δ-changes (in bold).

**Table 4:** Levels of and changes in health habits, body measurements, spirometry, blood pressure, quality of life and anxiety of 88 patients with T2DM randomized to intervention (n = 42) and control (n = 46) groups, from the beginning to eight months of this study.

		Groups			Cohen’s *d* Effect Size
		Intervention	Control	*p*-Value	
		Mean ± Stand. Dev.			
**Night-time sleep** (hours)	beginning	6.36 ± 1.84	6.50 ± 1.58		
	8 months	7.10 ± 1.38	6.03 ± 1.72		
	**Δ-change**	**0.74**	**−0.47**	<0.001	0.91
	Δ%-change	11.6	−7.3		
**Moderate-intensity activities in the last 7 days** (days)	beginning	1.69 ± 2.04	1.26 ± 1.98		
	8 months	2.25 ± 2.11	2.05 ± 1.99		
	**Δ-change**	**0.56**	**0.79**	0.586	0.12
	Δ%-change	33.1	62.9		
**Body mass index** (kg/m^2^)	beginning	31.36 ± 4.97	30.60 ± 5.49		
	8 months	30.89 ± 5.00	30.30 ± 5.22		
	**Δ-change**	**−0.47**	**−0.30**	0.463	0.16
	Δ%-change	−1.5	−1.0		
**Waist circumference** (cm)	beginning	108.64 ± 12.01	108.39 ± 10.82		
	8 months	107.76 ± 13.82	107.94 ± 10.54		
	**Δ-change**	**−0.88**	**−0.46**	0.704	0.08
	Δ%-change	−0.8	−0.4		
**Forced expiratory volume in the first second,** FEV_1_ (L)	beginning	2.18 ± 0.72	2.08 ± 0.61		
	8 months	2.30 ± 0.69	2.04 ± 0.61		
	**Δ-change**	**0.12**	**−0.03**	0.002	0.70
	Δ%-change	5.6	−1.5		
**Forced expiratory volume in the sixth second,** FEV_6_ (L)	beginning	2.64 ± 0.84	2.52 ± 0.73		
	8 months	2.91 ± 0.89	2.62 ± 0.82		
	**Δ-change**	**0.27**	**0.10**	0.055	0.42
	Δ%-change	10.1	4.1		
**Systolic blood pressure** (mmHg)	beginning	130.83 ± 14.08	129.85 ± 16.84		
	8 months	124.29 ± 13.66	129.61 ± 16.58		
	**Δ-change**	**−6.55**	**−0.24**	0.029	0.47
	Δ%-change	−5.0	−0.2		
**Diastolic blood pressure** (mmHg)	beginning	75.40 ± 8.97	75.76 ± 10.41		
	8 months	77.14 ± 8.91	76.87 ± 8.84		
	**Δ-change**	**1.74**	**1.11**	0.741	0.07
	Δ%-change	2.3	1.5		
**Physical component of quality of life** (SF-36 Scale) ^a^	beginning	68.69 ± 19.48	65.24 ± 20.02		
	8 months	82.68 ± 12.72	54.54 ± 20.88		
	**Δ-change**	**13.99**	**−10.71**	<0.001	1.58
	Δ%-change	20.4	−16.4		
**Mental component of quality of life** (SF-36 Scale) ^a^	beginning	67.13 ± 27.20	67.19 ± 22.66		
	8 months	81.47 ± 15.97	54.22 ± 26.05		
	**Δ-change**	**14.33**	**−12.97**	<0.001	1.46
	Δ%-change	21.4	−19.3		
**Anxiety** (SAST-10 Scale) ^b^	beginning	21.17 ± 3.16	21.35 ± 4.08		
	8 months	15.62 ± 4.16	22.04 ± 5.51		
	**Δ-change**	**−5.55**	**0.70**	<0.001	1.40
	Δ%-change	−26.2	3.3		

Student’s *t*-tests in Δ-changes (in bold). ^a^ Score 0–100 (higher indicates better quality of life). ^b^ Score 10–40 (higher indicates higher anxiety).

## Data Availability

The principal investigators maintained full access to the trial dataset, which was securely stored on password-protected servers at the University of Crete under strict access controls. External researchers may be granted access to the de-identified dataset upon reasonable request and following the implementation of appropriate data sharing agreements. To protect participant confidentiality, any personally identifiable information will be removed prior to data release. Anonymized results will be disseminated through presentations at international conferences and publications in peer-reviewed journals. Trial Registration Number: ISRCTN13131584.
